# MRI-Based Radiomic Signature Identifying Secondary Loss of Response to Infliximab in Crohn's Disease

**DOI:** 10.3389/fnut.2021.773040

**Published:** 2022-01-03

**Authors:** Jing Feng, Qi Feng, Yueying Chen, Tian Yang, Saiming Cheng, Yuqi Qiao, Jun Shen

**Affiliations:** ^1^Key Laboratory of Gastroenterology and Hepatology, Department of Gastroenterology and Hepatology, Inflammatory Bowel Disease Research Center, Ministry of Health, Shanghai, China; ^2^Ren Ji Hospital, Shanghai Jiao Tong University School of Medicine, Shanghai, China; ^3^Department of Gastroenterology and Hepatology, Shanghai Institute of Digestive Disease, Shanghai, China; ^4^Department of Radiology, Ren Ji Hospital, Shanghai Jiao Tong University School of Medicine, Shanghai, China

**Keywords:** Crohn's disease, radiomics, loss of response, infliximab, nomogram

## Abstract

Up to 50% of patients with Crohn's disease (CD) experience secondary loss of response (SLR) to infliximab. Patients with SLR may show clinical signs of iron deficiency as a result of inflammation despite being iron-replete. The magnetic resonance imaging (MRI)-based radiomic index, R2^*^, can detect changes in iron metabolism. Therefore, the R2^*^ parameter has considerable potential for detection of SLR to infliximab. The aims of this study were to explore the correlation between R2^*^ and inflammation and to develop a non-invasive nomogram based on R2^*^ to identify SLR to infliximab in patients with CD. Three hundred and twenty-two infliximab-treated patients with CD who underwent magnetic resonance enterography within 2 weeks before or after 54 weeks of infliximab therapy were divided into training and validation datasets at a ratio of 8:2. Point-biserial analysis was conducted to confirm the relationship between R2^*^ and inflammation. A multivariate logistic regression model was created using R2^*^, CRP and hemoglobin (OR, 1.10, 1.04 and 0.98; *P* < 0.05). Receiver-operating characteristic curves and the Hosmer-Lemeshow test were used to assess the performance of the model. A correlation between R2^*^ and inflammation was identified. Different trends in R2^*^ and iron status indices were observed between patients with responsive and non-responsive CD, which is worthy of further study. The model was converted to a visualized nomogram that had a good ability to discriminate the outcomes of infliximab therapy with an area under the curve of 0.723 (95% CI, 0.661–0.785) in the training dataset and 0.715 (95% CI, 0.587–0.843) in the validation dataset. We confirmed a correlation between R2^*^ and inflammation in patients with CD. Based on the MRI-based radiomic signature, a novel nomogram was established and validated to facilitate individualized identification of SLR to infliximab in patients with CD.

## Introduction

Crohn's disease (CD), a major subtype of inflammatory bowel disease (IBD), is a chronic remitting and relapsing inflammatory disorder of the gastrointestinal tract that requires lifelong treatment and follow-up (1). Biologic therapies, including anti-tumor necrosis factor (TNF), are effective in inducing and maintaining remission (2, 3); however, up to 50% of patients with an initial clinical response stop therapy due to secondary loss of response (SLR) (4). Loss of response to infliximab, an anti-TNF monoclonal antibody, is thought to emerge when drug levels become insufficient due to degradation and elimination of the anti-drug antibody. When loss of response to infliximab is suspected, an assessment for active inflammation and/or complications of IBD should be initiated, combined with evaluation of clinical manifestations, biochemical markers, endoscopy, and imaging. Moreover, routine monitoring of disease activity is necessary during infliximab therapy so that treatment options can be adjusted in a timely manner to ensure long-term remission.

For decades, the Crohn's Disease Activity Index (CDAI) has been considered a useful standard for assessing the clinical activity of CD (5–8). However, CDAI needs to be calculated over a period of 7 days, and correct calculation depends on good patient compliance, which limits its practicality for routine clinical application (9, 10). Colonoscopy is an objective and reliable method that reflects the status of the intestinal tract but is invasive, costly, and accompanied by risks of perforation, bleeding, and sedation-related cardiovascular complications (11). Moreover, endoscopy is not feasible in a significant proportion of patients with CD because of luminal stricture in the intestine and/or a penetrating phenotype of disease behavior. Moreover, repeated invasive operations require a longer recovery time and are psychologically burdensome for patients (12). Magnetic resonance enterography (MRE) is a non-invasive tool for comprehensive radiological assessment of disease activity, especially for patients who cannot be evaluated by endoscopy due to penetrating and stenotic lesions (13). The magnetic resonance index of activity (MaRIA), which is based on features such as wall thickness and mucosal ulceration, is widely used for objective evaluation of the activity of CD (14). However, use of the MaRIA requires simultaneous evaluation of a series of indicators, which is time-consuming and highly dependent on the competence of the radiologist.

To date, there has been no MRI-based radiomics signature that can identify disease activity in CD. However, detailed observation of MRI-based radiomic signatures suggests that the R2^*^ relaxation rate of liver tissue in gradient-echo images could be an indicator of CD status. R2^*^, known as the reciprocal of T2^*^, is directly proportional to iron and demonstrate the most promising result in evaluating iron deposition in the liver (15). And so R2^*^ can detect changes in iron metabolism, a characteristic of inflammation, which is facilitated by the presence of binding sites for proinflammatory cytokines in the promoter of genes regulating iron homeostasis (16). Moreover, as a quantitative parameter, R2^*^ is more objective and less operator-dependent than MaRIA. The aims of this study were to explore the role of R2^*^ in disease activity and to combine R2^*^ with clinical parameters to develop a non-invasive, sensitive, and convenient tool for identification of SLR to infliximab in patients with CD.

## Materials and Methods

### Study Design and Patients

This retrospective single-center study was conducted at the Ren Ji Hospital, Shanghai Jiao Tong University School of Medicine. The study was approved by the Ren Ji Hospital Ethics Committee (approval number KY2019-039) and conducted in accordance with the Declaration of Helsinki. The need for informed consent was waived in view of the retrospective observational nature of the study. Patient confidentiality was ensured by protection of all personal information in the medical record system.

**Figure 1:**
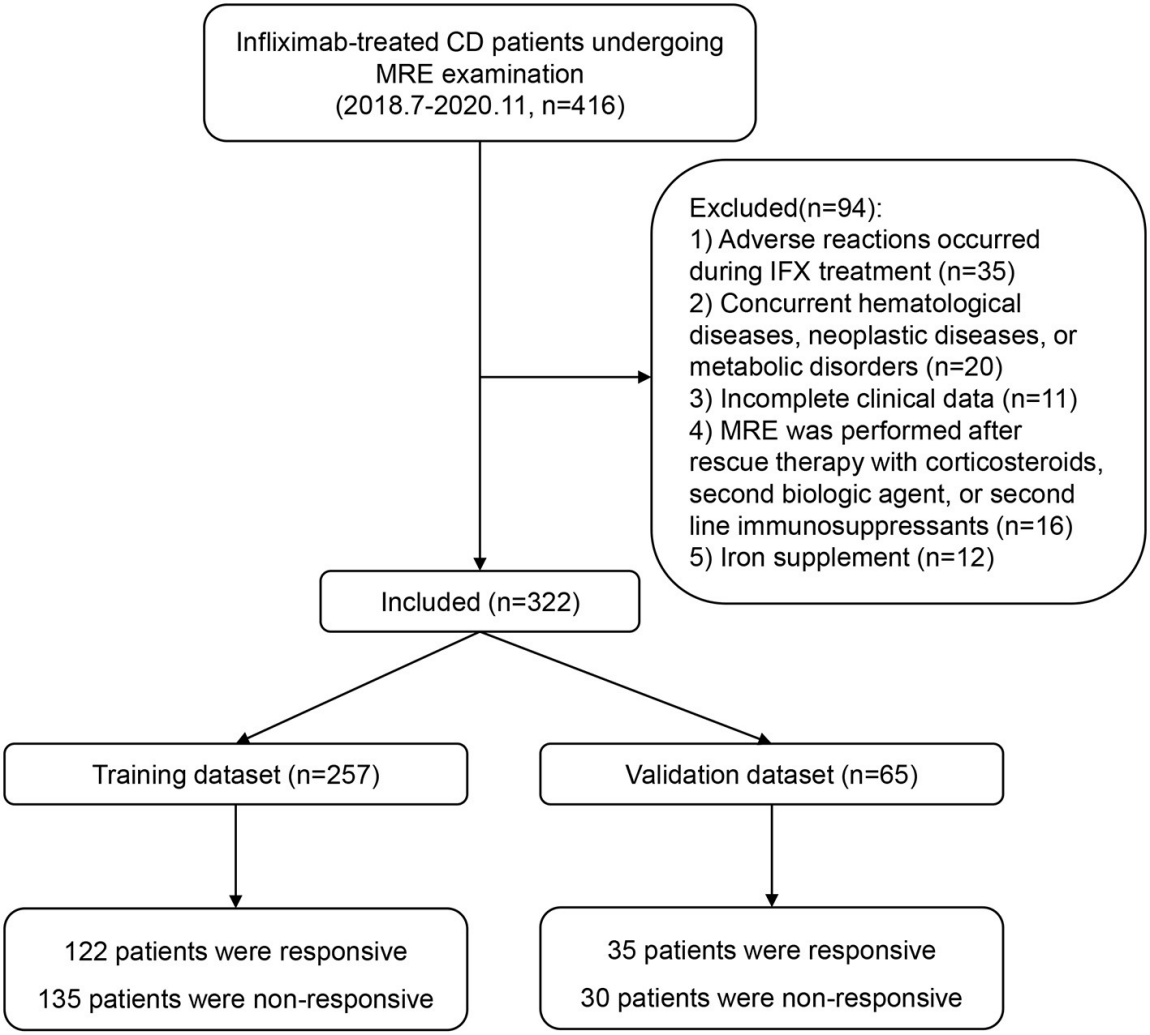
Recruitment process for patients in the training set and validation set.

Patients with CD who were treated with infliximab at Ren Ji Hospital between July 2018 and November 2020 were enrolled according to the eligibility criteria. The inclusion criteria were as follows: diagnosis of CD confirmed in accordance with the consensus statement published by the European Crohn's and Colitis Organization (17); treatment with infliximab initially effective and continued to 54 weeks or loss of response after week 14; MRE performed within 2 weeks before or after 54 weeks of infliximab therapy, or within 2 weeks after a relapse (CDAI >150); and a time interval between laboratory examinations and MRE of <1 week. The following exclusion criteria were applied: adverse reactions during infliximab treatment; concurrent hematological, neoplastic, metabolic, or auto-immune liver disease; incomplete clinical data; MRE performed after rescue therapy with corticosteroids, second biologic agents, or second-line immunosuppressants; and iron supplementation. A flowchart summarizing the details of the recruitment process is presented in Figure 1.

### MRE Image Acquisition and Calculation of R2^*^

Patients underwent bowel preparation 8 h before the MR examination. All patients were instructed to drink 1,000–1,200 mL of polyethylene glycol (Wanghe Pharma, Shenzhen, China) 45 min before scanning, and 10 mg of anisodamine (First Biochemical Pharma, Shanghai, China) was injected into the gluteus maximus muscle. MR examinations were performed on a 1.5-T GE scanner with an 8-channel torso phased-array coil (Optima MR360; GE Healthcare, Milwaukee, WI, USA). All images were obtained using routine MRE scanning in the supine position. In the contrast-enhanced phase, 15 mL of Magnevist (Bayer Schering, Shanghai, China) was injected through the dorsal vein.

The IDEAL-IQ sequence was used in this study. The R2^*^ map was generated using the post-processing software provided by the manufacturer. Five circular regions of interest (ROIs) of ~100 mm^2^ were drawn manually in the R2^*^ map using the AW4.6 workstation (GE Healthcare). The value of R2^*^ was displayed automatically once ROIs were drawn and the mean R2^*^ of five ROIs was considered as patient's R2^*^. Three of these ROIs were evenly placed on the right lobe and two on the left, avoiding major vessels, ligaments, and bile ducts. The ROI was delineated by two radiologists experienced in performing MRE and by an experienced gastroenterologist.

### Clinical Variables and Definition of Outcomes

Basic patient information was retrospectively extracted from the medical records. Information was collected on inflammation-related indicators, including the C-reactive protein (CRP) level and platelet count, and nutrition-related indicators, including hemoglobin, albumin, serum iron, mean erythrocyte hemoglobin concentration, mean red blood cell hemoglobin content, mean volume of hematocrit, total iron binding capacity, and transferrin. The Montreal classification was used to classify the location and behavior of the disease (18).

Secondary loss of response is defined in those patients who initially respond to infliximab therapy and subsequently lost clinical response (19). The CDAI value was used as a reference standard to determine whether patients responded to infliximab. The cut-off values were set at a CDAI score of ≤150 for inactive disease and >150 for active disease to indicate whether or not a patient showed a clinical response after treatment with infliximab. The SLR evaluation process was performed by a multi- disciplinary team of experienced experts at our institution during week 54. In brief, CDAI over 150 indicates loss of response. Subclinical recurrence would be determined by our MDT integrating the clinical symptoms, inflammatory biomarkers, endoscopic findings, and radiologic features. Patients with dose intensification or replacement with another anti-TNF drugs were also included as the secondary loss of response in this study.

### Construction and Validation of the Model

The enrolled patients were randomly divided into a training set and a validation set at a ratio of 8:2 using SPSS software (version 25.0; IBM Corp., Armonk, NY, USA). This ratio is the one that is commonly used to divide training and validation datasets (20–22). Univariate analysis was conducted to identify meaningful candidate variables for the model. Variables with a *P* < 0.2 in univariate analysis were considered potential candidates for further selection (23). Point-biserial analysis was conducted to further examine the correlation between the candidate variables and disease activity. Multivariate logistic regression analyses were performed with three selection procedures (forward, backward, and stepwise) to select the best-fit model based on the value of the Akaike information criterion. A nomogram was formulated from the results of the multivariate logistic analysis using the rms package in R (version 4.0.3; R Foundation for Statistical Computing, Vienna, Austria).

Receiver-operating characteristic (ROC) curve analysis was used to validate the performance of the model in both the training and validation sets. The calibration curve was evaluated using the Hosmer- Lemeshow goodness-of-fit test.

### Statistical Analysis

The statistical analysis was performed using R version 4.0.3 and STATA 15.0 for Windows (StataCorp LLC, College Station, TX, USA). Normally distributed continuous variables are expressed as the mean (standard deviation) and non-normally distributed variables as the median (interquartile range). Categorical variables are expressed as the proportion. Univariate analysis was performed using the Mann-Whitney *U*-test for continuous variables and the chi-squared test for categorical variables. Point-biserial analysis was conducted to identify the correlation between continuous and binary variables. The cut-off value of R2^*^ was calculated using the pROC package in R version 4.0.3. Logistic regression analysis was used to examine associations and find the best combination of variables.

Discrimination was quantified using the area under the ROC curve. Odds ratios with 95% confidence intervals (CIs) were calculated for the final predictors. The Hosmer-Lemeshow goodness of fit test was used to evaluate the model fit and a *P* > 0.05, indicating a good estimation of the model (24). All statistical tests were two-sided. A *P* < 0.05 was considered statistically significant.

## Results

### Demographics and Clinical Characteristics

A total of 322 patients who were treated with infliximab between July 2018 and November 2020 were recruited and subsequently randomized to a training set (*n* = 257) or an independent validation set (*n* = 65). The continuous variable hemoglobin was converted into two categories at a threshold of 120 g/L. The demographics and clinical characteristics of the patients in the training and validation sets are summarized in Table 1. In the training set, 122 patients (47.5%) showed an SLR to infliximab and recurrent inflammation, namely active CD, and 135 (52.5%) continued to respond to infliximab and remained in clinical remission. In the validation set, 35 patients (53.8%) had active CD and 30 (46.2%) had inactive CD. There were no statistically significant demographic or clinical differences between the two sets.

**Table 1:** Patient demographics and clinical characteristics.

**Characteristics**	**Training (*n* = 257)**	**Validation (*n* = 65)**	***P***
Age (y)	29.0 (22.0, 38.0)	28.0 (20.0, 34.0)	0.40
Gender			0.88
Male	181 (70.4%)	47 (72.3%)	
Female	76 (29.6%)	18 (27.7%)	
BMI (kg/m^2^)			0.92
<18.5	84 (32.7%)	20 (30.8%)	
18.5–24.9	145 (56.4%)	37 (56.9%)	
≥24.9	28 (10.9%)	8 (12.3%)	
Disease behavior			0.35
B1	148 (57.6%)	42 (64.6%)	
B2	62 (24.1%)	16 (24.6%)	
B3	47 (18.3%)	7 (10.8%)	
Perianal lesions	161 (62.6%)	43 (66.2%)	0.67
Hb (g/L)	137.0 (120.0, 149.0)	142.0 (122.0, 152.0)	0.33
R2*	27.1 (23.6, 31.8)	26.4 (23.2, 30.7)	0.30
MCHC (g/L)	332.0 (322.0, 339.0)	329.0 (317.0, 339.0)	0.26
MCV (fL)	88.5 (85.3, 91.5)	87.0 (82.3, 90.1)	0.12
MCH (pg)	29.6 (28.1, 30.7)	29.0 (27.1, 30.0)	0.11
PLT (*10∧9/L)	246.0 (209.0, 314.0)	245.0 (203.0, 291.0)	0.83
CRP (mg/L)	0.6 (0.5, 4.0)	1.3 (0.5, 9.8)	0.17
ALB (g/L)	44.7 (41.3, 47.9)	44.6 (41.1, 47.7)	0.71
Serum Iron (μmol/L)	12.4 (7.9, 17.1)	11.9 (6.3, 15.6)	0.22
TIBC (μmol/L)	54.1 (47.1, 60.4)	54.6 (50.2, 62.7)	0.31
Transferrin (g/L)	2.6 (2.2, 3.0)	2.7 (2.3, 2.9)	0.24
TS (%)	20 (10, 25)	20 (10, 25)	0.12

*Hb, hemoglobin; MCHC, mean erythrocyte hemoglobin concentration; MCV, mean volume of hematocrit; MCH, mean red blood cell hemoglobin content; PLT, platelet count; CRP, C-reactive protein; ALB, albumin; TIBC, total iron binding capacity; TS, transferrin saturation*.

### Contribution of Inflammation to Iron Utilization Disorders

**Figure 2:**
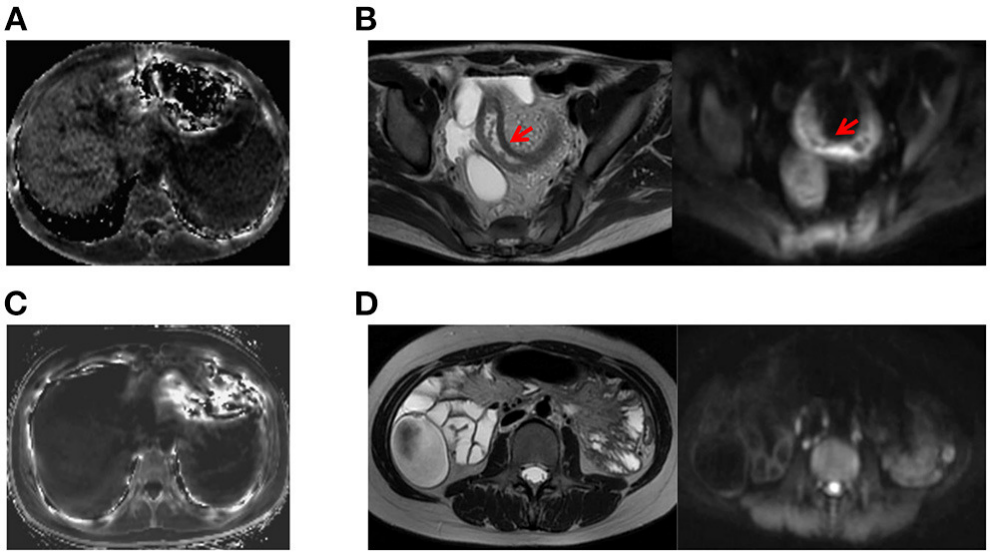
Examples of comparative MRE images obtained from patients with active CD and inactive CD. **(A)** A patient with active CD who showed higher liver signal intensity on the R2* map (R2* = 93.074; CDAI value, 233). **(B)** Corresponding T2-weighted and diffusion-weighted images of a patient with active CD showing inflammation of the bowel wall, including thickening of the wall of the ileum with an increased signal, rough mucosal surface, and a small amount of pelvic cavity effusion. **(C)** MRE image of a patient with inactive CD on the R2* map (R2* = 14.12, CDAI value, 73). **(D)** Corresponding T2-weighted and diffusion-weighted images for a patient with inactive CD. CD, Crohn's disease; CDAI, Crohn's Disease Activity Index; MRE, magnetic resonance enterography.

**Figure 3:**
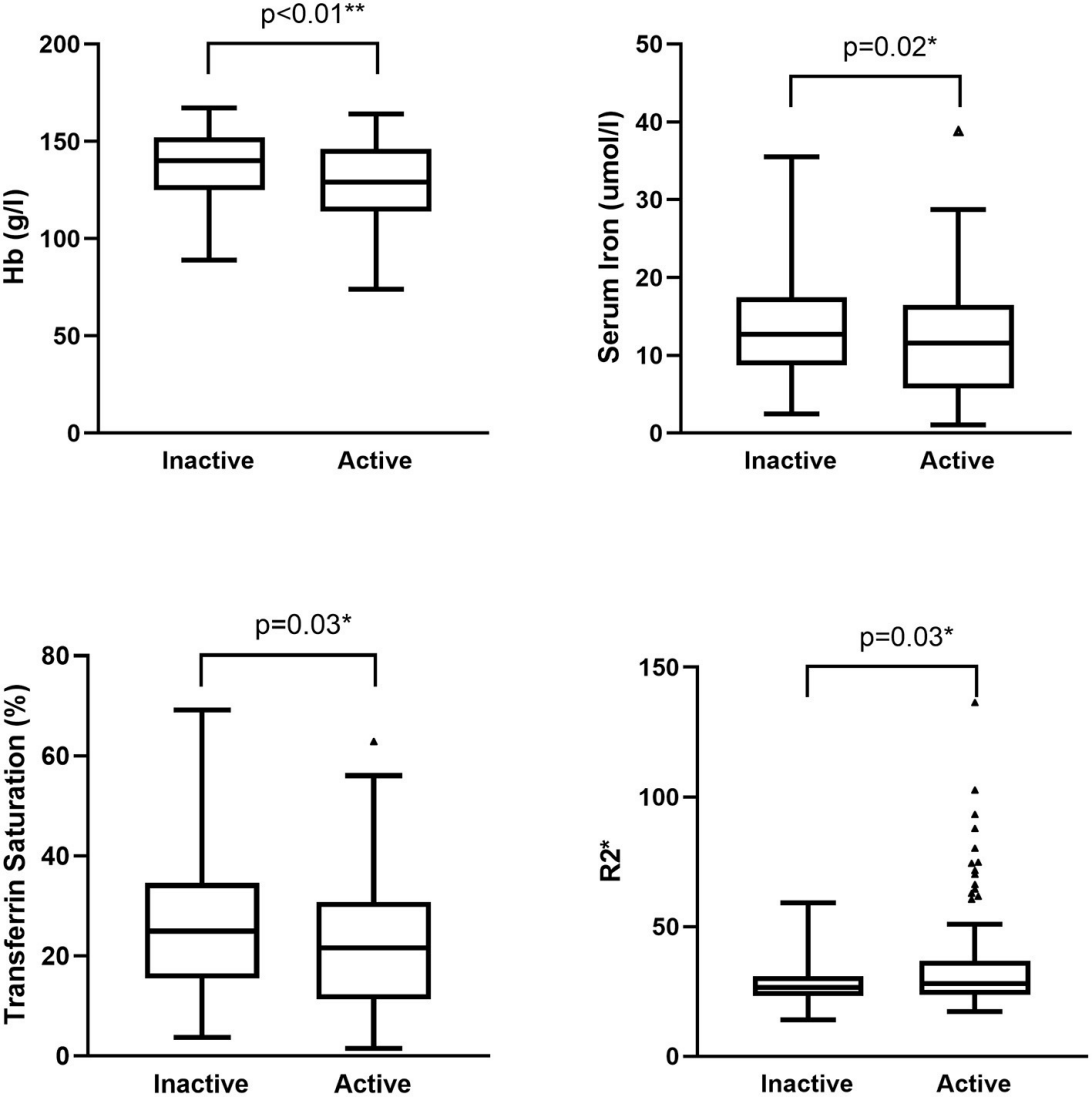
Difference in iron status indices and R2* between patients with active and inactive disease. Hemoglobin, serum iron, and transferrin saturation were higher in patients with inactive CD (*P* < 0.01, 0.02, and 0.03, respectively). R2* was higher in patients with active CD (*P* = 0.03). CD, Crohn's disease.

MR images of the infliximab-treated patients are shown in Figure 2. The R2^*^ map (Figures 2A,C) shows that there was a significant difference in signal intensity in the liver between patients with and without a clinical response to infliximab. Furthermore, the R2^*^ was significantly higher in patients with active disease (${\text{R2}}_{\text{active}}^{*}$ vs. ${\text{R2}}_{\text{inactive}}^{*}$ = 28.0 vs. 26.6, *P* = 0.03) whereas iron status indices showed an opposite trend (Figure 3). The outcome of point-biserial analysis showed that R2^*^ was significantly correlated with SLR (*r*_*p*_= 0.242, *P* < 0.001). The correlation between SLR and hemoglobin (*r*_*p*_= −0.242), serum iron (*r*_*p*_= −0.142), and transferrin saturation (*r*_*p*_= −0.139) was converse to that of R2^*^ and was statistically significant (all *P* < 0.05) (Table 2). R2^*^ was positively correlated with hepatic iron and outcomes of therapy, reflecting the difference in intestinal inflammation (15). The different trends in R2^*^ and iron status indices indicated that iron stores were adequate in patients with active CD but that recurrent intestinal inflammation might contribute to iron utilization disorders, resulting in iron deficiency (26). The possible cut-off value of R2^*^ is 35.655 with a lower AUC of 0.630 (Supplementary Figure 1), indicating that R2^*^ alone is not much practical for the identification of SLR.

**Table 2:** Correlations of secondary loss of response with R2^*^ and several iron status indices.

	***r_***p***_***	***p***
Hb	−0.242	<0.001
serum iron	−0.142	0.023
TS	−0.139	0.026
R2*	0.242	<0.001

*r_p_, correlation coefficient for Point-biserial analysis; Hb, hemoglobin; TS, transferrin saturation*.

### Development and Validation of a Clinical-Radiomics Nomogram to Identify SLR to Infliximab

**Figure 4:**
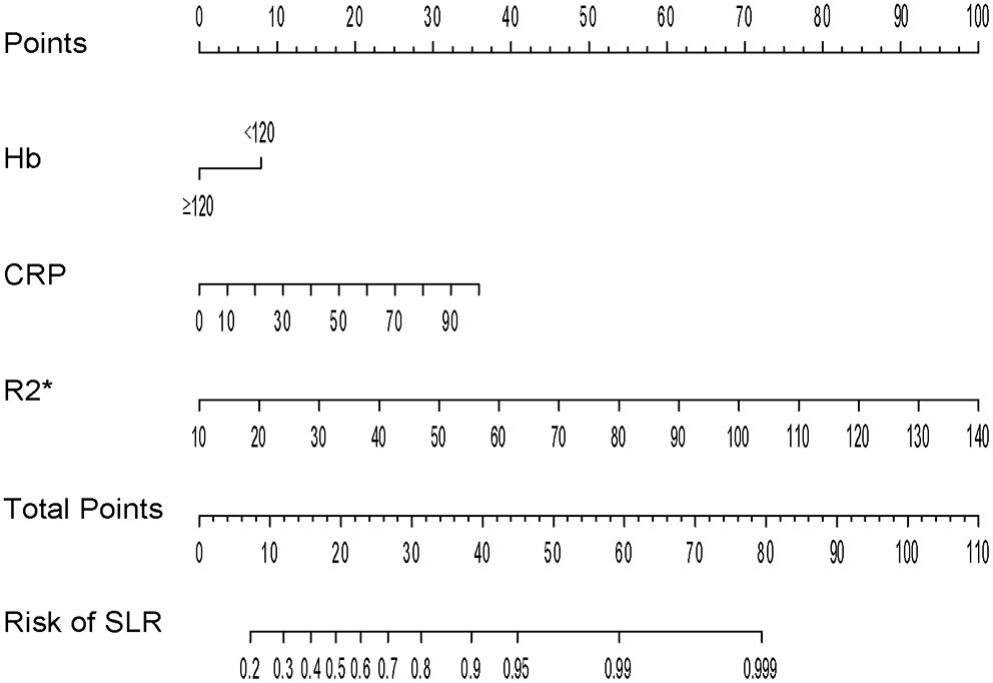
Nomogram for identification of secondary loss of response in patients with Crohn's disease.

For better identification, we used logistic regression to develop a new model based on R2^*^ and other clinical parameters. As shown in Table 3, variables with a *P* < 0.2 in univariate analysis, such as body mass index, hemoglobin, R2^*^, and CRP level, were considered as candidate variables. The remaining variables were not significantly associated with SLR in our training cohort. Three selection procedures (forward, backward, and stepwise) were applied to further confirm the best-fit model; CRP, hemoglobin, and R2^*^ were finally determined as the appropriate subset of variables to identify SLR (odds ratios, 1.04, 0.98, and 1.10, respectively; *P* < 0.05). The diagnostic equation for the model was: 0.406 + 0.035^*^CRP - 0.025^*^Hb + 0.090^*^R2^*^. Specific information on the model is presented in Table 4. To further simplify the logistic regression results and to create a clinically practical tool, the coefficients of each factor in the multivariate logistic regression analysis were used as weights to construct a nomogram that would facilitate the practical application of the model for identifying the risk of SLR in a given patient (Figure 4).

**Table 3:** Results of univariate analysis of candidate variables for the training dataset.

**Variables**	**Inactive/responders (*n* = 135)**	**Active/non-responders (*n* = 122)**	***P***
Age (y)	28.0 (21.0, 37.0)	29.0 (22.0, 39.0)	0.36
Gender			0.79
Male	94 (69.6%)	87 (71.3%)	
Female	41 (30.4%)	35 (28.7%)	
BMI (kg/m∧2)			0.07
<18.5	36 (26.7%)	48 (39.3%)	
18.5–24.9	85 (63.0%)	60 (49.2%)	
≥24.9	14 (10.4%)	14 (11.5%)	
Disease behavior			0.33
B1	81 (60.0%)	67 (54.9%)	
B2	34 (25.2%)	28 (23.0%)	
B3	20 (14.8%)	27 (22.1%)	
Perianal lesions	87 (64.4%)	74 (60.7%)	0.61
Hb			<0.01
<120 g/L	20 (14.8%)	43 (35.2%)	
≥120 g/L	115 (85.2%)	79 (64.8%)	
MCHC (g/L)	335.0 (325.0, 341.0)	329.0 (319.0, 337.0)	<0.01
MCV (fL)	88.5 (85.9, 90.3)	88.4 (84.1, 92.3)	0.99
MCH (pg)	29.7 (28.4, 30.9)	29.5 (26.8, 30.5)	0.04
PLT (*10∧9/L)	239.0 (202.0, 288.0)	258.5 (215.0, 328.0)	0.02
CRP (mg/L)	0.6 (0.5, 1.8)	1.3 (0.5, 10.5)	0.02
ALB (g/L)	45.1 (42.4, 48.1)	44.3 (39.4, 47.8)	0.07
Serum iron (μmol/L)	12.7 (8.7, 17.5)	11.6 (5.8, 16.5)	0.02
TIBC (μmol/L)	54.7 (49.1, 60.4)	53.0 (44.5, 60.6)	0.26
Transferrin (g/L)	2.6 (2.3, 3.0)	2.5 (2.2, 2.9)	0.09
TS (%)	30 (20,30)	20 (10,30)	0.03
R2*	26.6 (23.4, 31.0)	28.0 (23.8, 36.6)	0.03

*Hb, hemoglobin; MCHC, mean erythrocyte hemoglobin concentration; MCV, mean volume of hematocrit; MCH, mean red blood cell hemoglobin content; PLT, platelet count; CRP, C-reactive protein; ALB, albumin; TIBC, total iron binding capacity; TS, transferrin saturation*.

**Table 4:** Variables in the final multi-regression model in the training dataset.

**Intercept and variable**	**β**	**Odds ratio (95% CI)**	***P***
Intercept	0.406	–	–
CRP	0.035	1.036 (1.001–1.072)	0.044
Hb	−0.025	0.976 (0.960–0.992)	0.003
R2*	0.090	1.094 (1.047–1.143)	<0.001
Area under ROC curve		–	
Training dataset		0.723(0.661–0.785)	
Validation dataset		0.715 (0.587–0.843)	

*CI, confidence interval; CRP, C-reactive protein, Hb, hemoglobin*.

**Figure 5:**
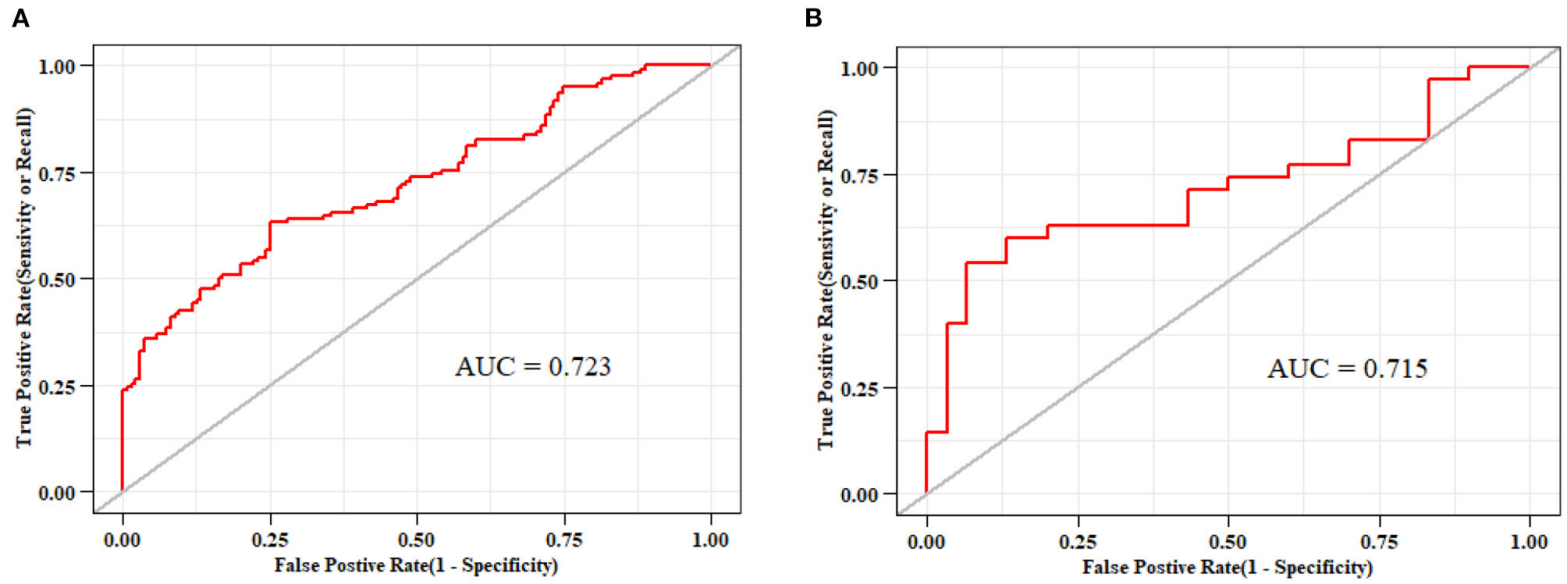
Receiver-operating characteristic curves for nomograms used to identify secondary loss of response to infliximab **(A)** in the training cohort (area under the curve, 0.723) and **(B)** validation cohort (area under the curve, 0.715).

The ROC curves of the nomogram in the training and validation datasets are shown in Figure 5. Briefly, the nomogram was equipped with satisfactory discrimination power to identify SLR; the area under the curve was 0.723 (95% CI, 0.661–0.785) in the training dataset and 0.715 (95% CI, 0.587–0.843) in the validation dataset. Furthermore, the nomogram was well-calibrated, with a Hosmer-Lemeshow c2 statistic of 6.98 (*P* = 0.73) in the training dataset and 13.12 (*P* = 0.22) in the validation dataset, demonstrating that its monitoring efficacy was highly consistent with that of the CDAI (Supplementary Figure 2).

## Discussion

Although CD can generally be well-controlled by anti-TNF therapy, up to 50% of patients may lose their initial therapeutic response over time. To achieve a better prognosis, it is imperative to objectively confirm SLR using a comprehensive assessment of CD activity. In the present study, we developed and validated a novel non-invasive, MRI-based robust radiomics nomogram for identification of SLR to infliximab in patients with CD. This study confirmed the value of R2^*^ when monitoring the outcomes in patients with CD treated with infliximab, highlighting a novel signature for monitoring the progression of CD. To the best of our knowledge, this is the first attempt to identify SLR to infliximab based on an MRI-based radiomics signature.

Radiomics is an emerging field of medical imaging and is typically used to quantify tumor heterogeneity (27–29). Inspired by the promising potential of radiomics, we sought to expand its use to non-neoplastic diseases and develop a clinical-radiomics model that allows more comprehensive assessment of SLR in patients with CD. Considering the radiation dose during computed tomography enterography, R2^*^, a radiomics signature based on MRI, was selected as the core for our model. In contrast with the complexity of MaRIA, R2^*^ can be obtained in a more rapid and less operator-dependent manner, making the model a more objective and practical tool. Moreover, MRE can be performed without being hindered by strictured bowel segments, which means that our model has a wider scope of applicability.

R2^*^ is commonly used to monitor iron overload and is positively related to the liver iron concentration. Although frank iron overload was not present in any individual in our study, R2^*^ was significantly higher in patients with non-responsive CD than in those with responsive disease. Patients with active CD, namely, non-responsive patients in whom inflammation of the intestinal mucosa did not achieve remission, had higher levels of hepatic iron. However, the difference in R2^*^ between patients with and without active CD appears to be contradictory to that of the iron status indices. Comparative analysis revealed that the values for iron status parameters, including serum iron, transferrin, and hemoglobin, were lower in patients with active disease, which has previously been attributed mainly to iron deficiency anemia as a result of chronic intestinal blood loss or decreased iron absorption in the damaged duodenum (25, 30). However, our findings with regard to R2^*^ show that patients with active CD have adequate hepatic iron, indicating that iron deficiency may not be the cause of the lower serum iron-related parameters in patients with CD and intestinal inflammation. A possible explanation for this might be impairment of iron utilization caused by recurrent inflammation and anemia of chronic disease (ACD).

The pathogenesis and specific mechanisms of ACD remain to be elucidated. The evidence suggests that hepcidin, a protein hormone synthesized mainly in hepatocytes, is a critical mediator of ACD and maintains iron homeostasis in patients with IBD (31). The increase in proinflammatory cytokines such as interleukin-6 during inflammation stimulates production of hepcidin in the liver. High levels of hepcidin could lead to a defect in the ability of enterocytes to transport iron and an inability of reticuloendothelial cells to recycle iron, resulting in development of ACD (32). This suggestion is in agreement with our findings and could explain why a proportion of patients with active CD show symptoms of anemia but have an adequate (or even elevated) iron reserve. Our findings regarding R2^*^, which measures the iron concentration in the liver with MRE, reflect inflammatory factors contributing to anemia without the need for iron supplementation. Our nomogram and multivariate logistic regression analysis also found that CRP, hemoglobin should be included in the assessment of disease activity, which confirms the findings of previous studies (33). The relevant studies have usually combined several inflammatory indicators, including CRP and interleukin-6, to evaluate disease activity without modeling (34–36), whereas we combined inflammation with nutrition-related indicators to build a model, not only improving clinical efficiency but also corroborating the new insight that nutrition-related factors are environmental triggers for development and modification of lifestyle-related chronic diseases, including IBD (37).

Our study had several strengths. To the best of our knowledge, it is the first to report the importance of R2^*^ in the treatment of CD. The model we have built incorporated two major types of indicators, providing a new approach for identification of SLR. Another advantage of this study is that the model was translated into a visualized nomogram, making it an easy and convenient tool for clinical use. Moreover, the association between inflammation and iron was further confirmed, suggesting that patients with CD who show signs of iron deficiency do not necessarily require iron supplementation.

Nevertheless, the study also has several limitations. First, as a single-center retrospective study, most enrolled patients resided in the same area, and the observed effect might not fully represent the general patient population with CD. The verification of external data from different areas or hospitals is lacking. Second, the number of eligible patients was limited due to the low prevalence of CD and high cost of infliximab. The nomogram can only be internally validated in a relatively small cohort. It would be desirable to expand the sample size in future studies to improve the statistical power and expand the application range of the model. Third, the definition of clinical response to infliximab in this study was based on the CDAI value, given that some of the patients had penetrative and/or obstructive phenotypes, which were contraindications to endoscopic evaluation. Whether the nomogram can reliably evaluate underlying mucosal healing must be addressed further. Besides, pre-treatment data of R2^*^ was not available in this study. The correlation between R2^*^ and severity of intestinal inflammation needs to be researched in depth with both pre- and post-treatment data.

In conclusion, this study has defined the association between R2^*^ and disease activity in patients with CD. The possible underlying mechanism provides a feasible solution to determine the effect of the inflammatory state on iron metabolism. An innovative nomogram was established and validated based on an MRI-based radiomic signature to facilitate individualized identification of SLR to infliximab in patients with CD. Nevertheless, large-scale prospective studies should be conducted to further enhance the accuracy and applicability of the model.

## Data Availability Statement

The raw data supporting the conclusions of this article will be made available by the authors, without undue reservation.

## Ethics Statement

Written informed consent was obtained from the individual(s) for the publication of any potentially identifiable images or data included in this article.

## Author Contributions

JF collected and analyzed clinical data. YC and TY helped collect part of the clinical data. QF and SC collected and analyzed radiological data. JF and QF drafted the manuscript. YQ presented the idea of this paper. QF and JS supported the funding. YQ and JS analyzed the conclusions, edited, and revised the manuscript. All authors contributed to the article and approved the submitted version.

## Funding

This work was supported by grants from National Natural Science Foundation of China (Nos. 81770545 and 81701746), MDT Project of Clinical Research Innovation Foundation, Ren Ji Hospital, and Shanghai Jiao Tong University School of Medicine (PYI-17-003).

## Conflict of Interest

The authors declare that the research was conducted in the absence of any commercial or financial relationships that could be construed as a potential conflict of interest.

## Publisher's Note

All claims expressed in this article are solely those of the authors and do not necessarily represent those of their affiliated organizations, or those of the publisher, the editors and the reviewers. Any product that may be evaluated in this article, or claim that may be made by its manufacturer, is not guaranteed or endorsed by the publisher.
