# Supplementary material for: MRI-Based Radiomic Signature Identifying Secondary Loss of Response to Infliximab in Crohn's Disease
Source: Front Nutr. 2022 Jan 3;8:773040. doi: 10.3389/fnut.2021.773040 (PMC8763017; doi:10.3389/fnut.2021.773040)

Supplementary Material

# Supplementary Figures

**Supplementary Figure 1.** The ROC curves of R2* to identify SLR (AUC=0.630).

**
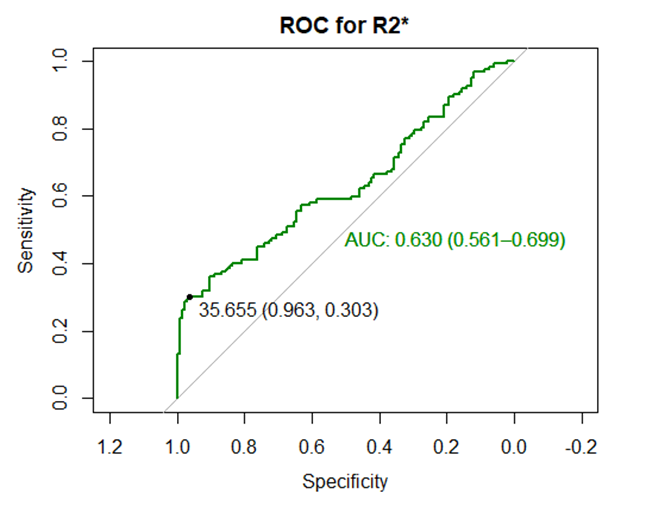
**

**Supplementary Figure 2.** Calibration curve for nomograms used to identify secondary loss of response to infliximab in the (A) training cohort (P=0.73) and (B) validation cohort (P=0.22).


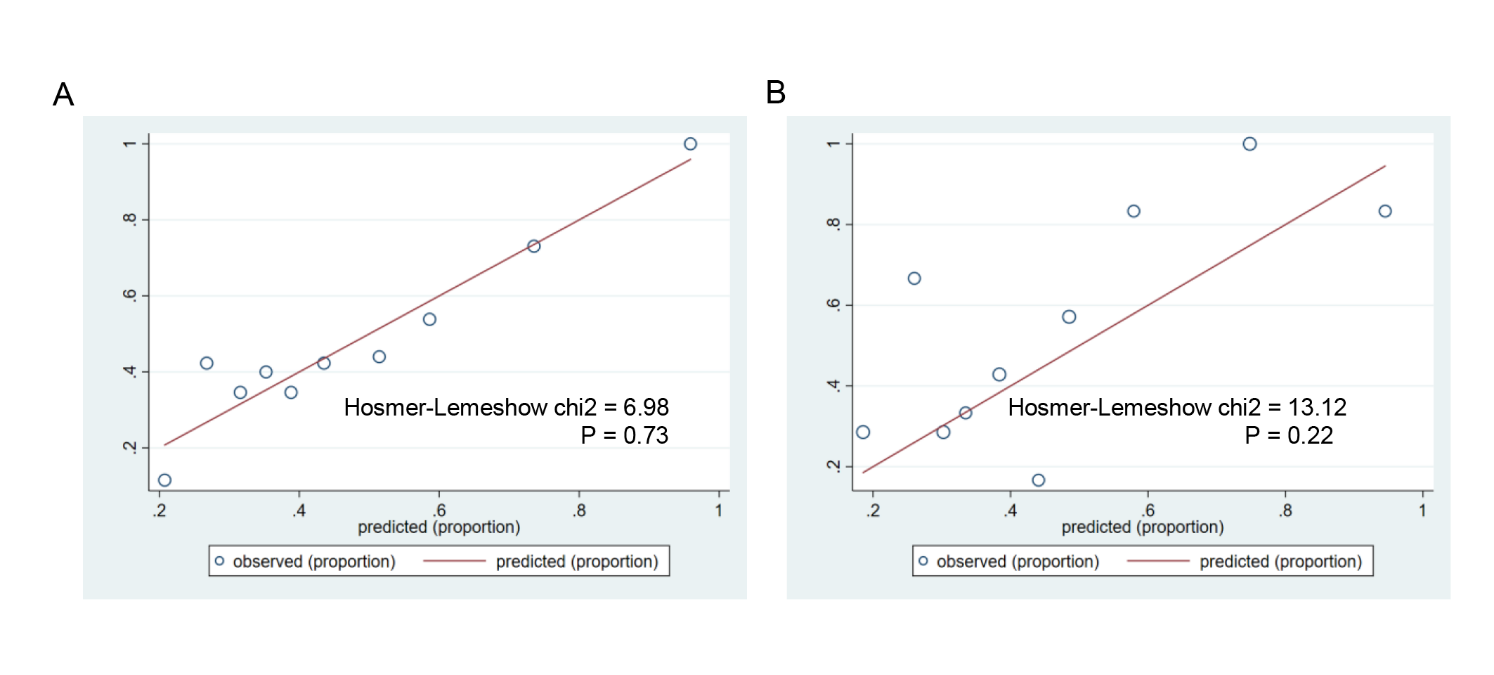

Supplement: Supplementary file 1 [file Table_1.DOCX]
